# Pre-Operative Versus Post-Operative Radiosurgery of Brain Metastases—Volumetric and Dosimetric Impact of Treatment Sequence and Margin Concept

**DOI:** 10.3390/cancers11030294

**Published:** 2019-03-01

**Authors:** Rami A. El Shafie, Eric Tonndorf-Martini, Daniela Schmitt, Dorothea Weber, Aylin Celik, Thorsten Dresel, Denise Bernhardt, Kristin Lang, Philipp Hoegen, Sebastian Adeberg, Angela Paul, Jürgen Debus, Stefan Rieken

**Affiliations:** 1 Department of Radiation Oncology, University Hospital of Heidelberg, Im Neuenheimer Feld 400, 69120 Heidelberg, Germany; eric.tonndorf-martini@med.uni-heidelberg.de (E.T.-M.); daniela.schmitt@med.uni-heidelberg.de (D.S.); aylin_ck@hotmail.de (A.C.); dreselthorsten@web.de (T.D.); denise.bernhardt@med.uni-heidelberg.de (D.B.); kristin.lang@med.uni-heidelberg.de (K.L.); philipp.hoegen@med.uni-heidelberg.de (P.H.); sebastian.adeberg@med.uni-heidelberg.de (S.A.); angela.paul@med.uni-heidelberg.de (A.P.); juergen.debus@med.uni-heidelberg.de (J.D.); stefan.rieken@med.uni-heidelberg.de (S.R.); 2 National Center for Radiation Oncology (NCRO), Heidelberg Institute for Radiation Oncology (HIRO), Im Neuenheimer Feld 400, 69120 Heidelberg, Germany; 3 Institute of Medical Biometry and Informatics (IMBI), University of Heidelberg, Im Neuenheimer Feld 130.3, 69120 Heidelberg, Germany; weber@imbi.uni-heidelberg.de; 4 Heavy Ion Therapy Center (HIT), Heidelberg University Hospital, Im Neuenheimer Feld 450, 69120 Heidelberg, Germany; 5 Clinical Cooperation Unit Radiation Oncology (E050), German Cancer Research Center (DKFZ), Im Neuenheimer Feld 280, 69120 Heidelberg, Germany

**Keywords:** tumor, radiosurgery, neurosurgery, metastases, radiotherapy, radiation therapy, stereotactic

## Abstract

Background: Pre-operative radiosurgery (SRS) preceding the resection of brain metastases promises to circumvent limitations of post-operative cavity SRS. It minimizes uncertainties regarding delineation and safety margins and could reduce dose exposure of the healthy brain (HB). Methods: We performed a systematic treatment plan comparison on 24 patients who received post-operative radiosurgery of the resection cavity at our institution. Comparative treatment plans were calculated for hypofractionated stereotactic radiotherapy (7 × 5 Gray (Gy)) in a hypothetical pre-operative (pre-op) and two post-operative scenarios, either with (extended field, post-op-E) or without the surgical tract (involved field, post-op-I). Detailed volumetric comparison of the resulting target volumes was performed, as well as dosimetric comparison focusing on targets and the HB. Results: The resection cavity was significantly smaller and different in morphology from the pre-operative lesion, yielding a low Dice Similarity Coefficient (DSC) of 53% (*p* = 0.019). Post-op-I and post-op-E targets showed high similarity (DSC = 93%), and including the surgical tract moderately enlarged resulting median target size (18.58 ccm vs. 22.89 ccm, *p* < 0.001). Dosimetric analysis favored the pre-operative treatment setting since it significantly decreased relevant dose exposure of the HB (Median volume receiving 28 Gy: 6.79 vs. 10.79 for pre-op vs. post-op-E, *p* < 0.001). Dosimetrically, pre-operative SRS is a promising alternative to post-operative cavity irradiation that could furthermore offer practical benefits regarding delineation and treatment planning. Comparative trials are required to evaluate potential clinical advantages of this approach.

## 1. Introduction

Neurosurgical resection is recommended by current international guidelines for large, symptomatic brain metastases (BM), or for the procurement of a pathologic sample [1,2]. There exists ample evidence that post-operative radiotherapy (RT) can improve local control while probably not directly affecting overall survival [3,4,5,6]. Post-operative whole-brain irradiation (WBRT) has been proved effective and has represented the established standard of care for several decades [4,5]. However, the position of WBRT has been challenged by the increasing use of more precise modalities such as single-fraction stereotactic radiosurgery (SRS) or hypofractionated stereotactic radiotherapy (HFSRT). This was recently demonstrated by two phase 3 trials, concluding that SRS improves local control over observation, as reported by Mahajan et al., while significantly reducing neurocognitive toxicity when compared to WBRT, as reported by Brown et al. [6,7].

While omitting WBRT in favor of SRS could lead to an increased risk of recurrence in the untreated brain, those recent phase 3 trials surprisingly showed inferior local control after SRS at the resection cavity as well, as compared to WBRT. To date, the reasons for this finding are inconclusive, though two major hypotheses have been discussed. Firstly, the small safety margins of 1–2 mm employed in the trials might not have adequately accounted for microscopic tumor infiltration. Secondly, the applied dose was dependent on cavity size, allowing for dose reduction down to 12 Gray (Gy) for large cavities. It can thus be argued that a relevant number of patients were treated with a dose possibly insufficient for lasting tumor control [6,7].

Pre-operative SRS preceding brain metastasis resection is an alternative approach that could help circumvent uncertainties regarding margins and target delineation [8]. Morphological changes of the resection cavity increase those uncertainties in the post-operative setting [9]. In contrast, by treating a pre-operative and thus plainly visible target, those morphological changes are rendered irrelevant. Further potential advantages to the pre-operative approach include the reduction of healthy brain tissue included in the target volume by the reduction or omission of safety margins and the lack of a resection tract [8]. This could lead to dosimetric advantages, reducing integral dose to the brain, and thus the overall risk of radio-necrosis [10]. It is furthermore discussed, if the risk of leptomeningeal dissemination could be reduced by pre-operative tumor cell sterilization [11].

Though the pre-operative approach has been proposed as an alternative to post-operative SRS, comparative clinical data is scarce [8]. Furthermore, the potential volumetric and dosimetric advantages are hypothesized but have yet to be conclusively demonstrated. It is the aim of this work to investigate the potential of pre-operative vs. post-operative SRS by providing a detailed volumetric and dosimetric comparison. To this end, we compared the pre-operative setting with two post-operative scenarios, using different contouring approaches—one targeting the involved field around the cavity and the other an extended field that includes the complete surgical tract.

## 2. Results

We identified 24 patients who presented for the post-operative irradiation of the resection cavity at our institution in 2016 and 2017. For each patient, we analyzed survival and local control following the actually performed treatment. In a second step, on the same series, we conducted a proof-of-concept volumetric and dosimetric experiment to simulate the effects of pre-operative SRS and the effects of different contouring approaches. To this end, we delineated a new set of target volumes and calculated three comparative treatment plans for dosimetric analysis.

### 2.1. Patient Characteristics and Performed Treatment

Median age at the beginning of treatment was 60 years (interquartile range (IQR): 52–64), and most common histology was non-small cell lung cancer (*n* = 9, 37.5%), followed by breast cancer (*n* = 7, 29.2%). Ten patients (41.7%) had additional unresected brain metastases that were treated with SRS. Detailed patient characteristics and a description of the treatment performed are illustrated in Table 1 and Table 2.

### 2.2. Survival and Local Control

Median follow-up, as calculated by the reverse Kaplan-Meier method, was 7.40 months (IQR: 1.77–11.97) for the endpoint of local recurrence and 12.90 months (IQR: 4.03–13.40) for the endpoint of death. At the time of analysis, five patients had died, and 19 patients were still alive. Two patients developed local progression at the irradiated resection cavity at 8.8 and 20.4 months from RT, respectively. In one case, local progression occurred multifocally at the resection margins of the cavity and in the surgical tract. In the other case, the surgical tract was not involved. In both cases, the surgical tract had been included in the initial target volume. Crude local progression-free survival rates at the irradiated cavity (cPFS) were 100% at six months and 95.8% at 12 months; median cPFS was not reached. Two patients developed radionecrosis at the irradiated resection cavity. One patient received SRS at a margin dose of 18 Gy. The other received FSRT at 6 × 5 Gy, although this latter patient had undergone incomplete resection and the residual macroscopic tumor was treated with an integrated boost up to 6 × 6 Gy. This patient also developed multifocal local failure later on. Crude PFS rate in the distant brain (dPFS) was 83.3% at six months and 70.8% at 12 months; median Kaplan-Meier estimate for dPFS was 13.13 months (IQR: 7.40–not reached). Median Kaplan-Meier estimate for overall survival (OS) in all treated patients was not reached (IQR: 10.0 months–not reached). OS was 83.3% at 6 months and 79.1% at 12 months. The most common pattern of distant brain failure was leptomeningeal dissemination (LMD), affecting five out of nine patients; four patients developed new distant BM. Three out of five patients developing LMD had breast cancer histology, the other two had gastrointestinal primaries.

### 2.3. Volumetric Analysis

**Figure 1:**
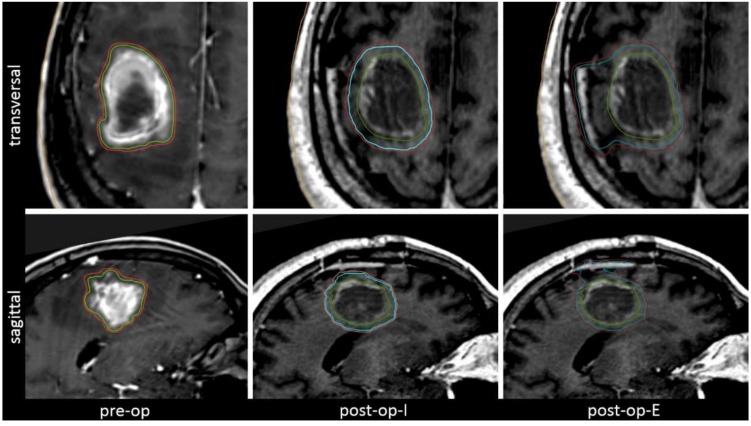
The three clinical scenarios regarded for volumetric and dosimetric analysis, illustrated by means of a representative example. The pre-operative (pre-op) setting includes only a minimal margin. The post-operative settings target either the involved field (cavity + 3 mm, post-op-I) or an extended field that includes the complete surgical tract (post-op-E).

The hypothetical scenarios used for comparative treatment plans included a pre-operative scenario with a 1 mm margin added to the visible tumor (pre-op) and two post-operative scenarios. The post-operative involved field-scenario (post-op-I) included a 3 mm margin added to the visible cavity and the post-operative extended-field scenario (post-op-E) additionally included the complete surgical tract. Further details on the treatment scenarios for comparative plan analysis are illustrated in Figure 1 and are described in the Methods and Materials Section.

**Figure 2:**
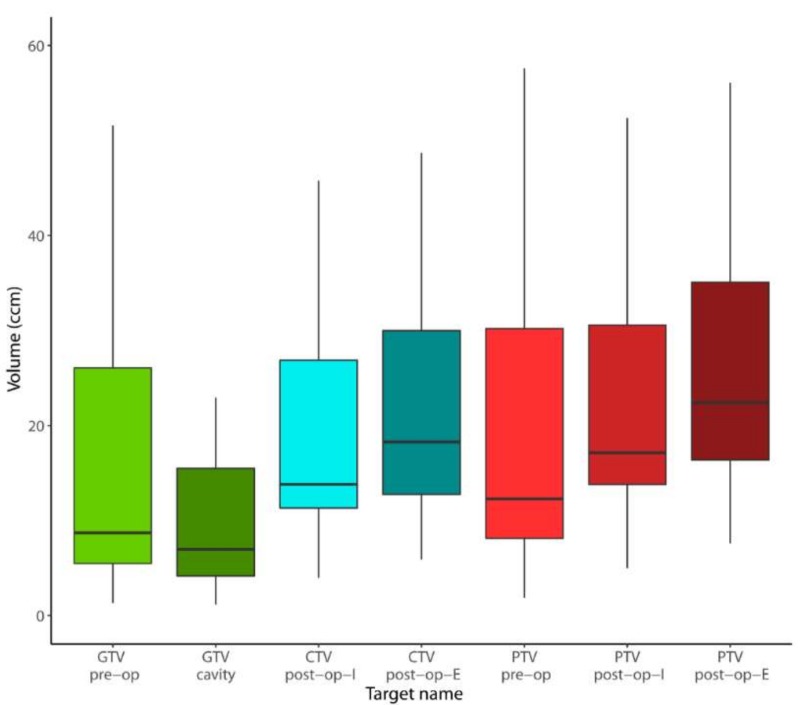
Boxplot illustrating target volume sizes in comparison. Abbreviations: GTV: gross target volume; CTV: clinical target volume; PTV: planning target volume; pre-op: pre-operative setting; post-op-I: post-operative involved field (cavity + 3 mm); and post-op-E: post-operative extended field (including surgical tract).

**Figure 3:**
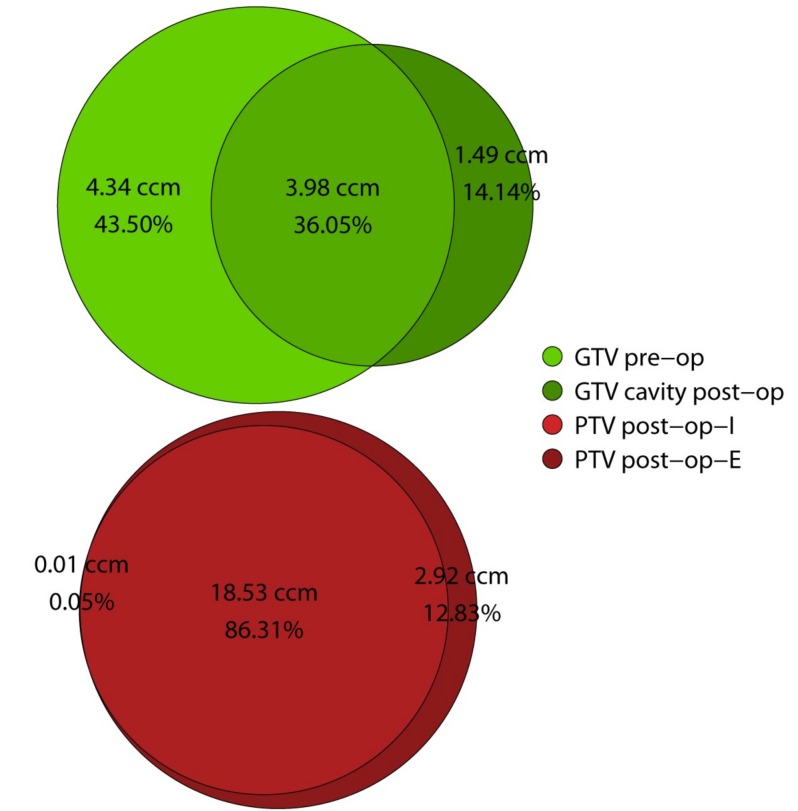
Venn diagram illustrating similarity, areas of overlap and difference between target volumes. Median absolute volumes (ccm) are indicated above; below are listed volumes relative to the combined sum of the two respective targets (%). Further details available in Table 3. Abbreviations: GTV: gross target volume; PTV: planning target volume; pre-op: pre-operative setting; post-op-I: post-operative involved field (cavity + 3 mm); and post-op-E: post-operative extended field (including surgical tract).

Median cavity volume was significantly smaller, compared to the size of the unresected metastasis (6.96 vs. 8.71 ccm, *p* = 0.019) (Figure 2). Additionally, the post-operative gross target volume (GTV) of the cavity differed remarkably from the pre-operative GTV, yielding a median Dice Similarity Coefficient of only 53% (IQR: 46–63%). The median overlap between pre- and post-op was 3.98 ccm (IQR: 2.31–13.69), representing only 36.05% (IQR: 29.46–46.26%) of the combined pre and post-operative GTVs. A median of 43.50% (IQR: 24.14–51.35%) of the combined GTVs were in the pre-operative but not in the post-operative GTV and conversely, 14.14% (IQR: 5.85–27.89%) of the combined GTVs were in the post-operative and not in the pre-operative GTV. Those findings indicate considerable morphological changes regarding form and location of the post-operative cavity when compared to the pre-operative anatomy (Figure 3).

In comparison, the final planning target volumes (PTV) resulting from the different contouring approaches in post-op-I and post-op-E showed reasonably high similarity, yielding a median DSC of 93% (IQR: 87–94%). The median overlap between post-op-I and post-op-E PTV was 86.31% (IQR: 77.45–89.27%]. Median PTV size at 22.89 ccm (IQR: 16.37–37.21) was significantly larger for post-op-E, as compared to 18.58 ccm (IQR: 13.88–31.30) for post-op-I (*p* = 0.016). This difference in volume translated into a median of 2.92 ccm (IQR: 2.14–4.84) of additional tissue included in the PTV in the post-op-E setting that was not included in the post-op-I setting. The difference makes up for 12.83% (IQR: 10.34–34.68%) of the combined post-op-I and post-op-E PTVs (Figure 3).

The absolute increase in target volume size attributable to the addition of the respective safety margins (total margin = PTV minus GTV) differed considerably between the three treatment scenarios. This volume increase amounted to a median of 2.47 ccm (IQR: 1.70–4.35) in the pre-op scenario, as compared to 12.14 ccm (IQR: 9.31–16.57) for post-op-I (*p* < 0.001) and 15.90 ccm (IQR: 11.52–22.87) for post-op-E (*p* < 0.001). Here again, the difference between post-op-I and post-op-E was significant (*p* < 0.001). Compared to the size of macroscopic tumor or cavity respectively (GTV), the addition of the respective safety margin increased the PTV by a median of 23.75% (IQR: 15.87–30.36%) in the pre-op scenario. In contrast, this median relative increase was 175.91% (IQR: 107.25–221.50%) in the post-op-I scenario and 224.41% (IQR: 139.49–290.27%) in the post-op-E scenario. Details on volumetric analysis are displayed in Table 3 and Table 4.

### 2.4. Dosimetric Analysis

**Figure 4:**
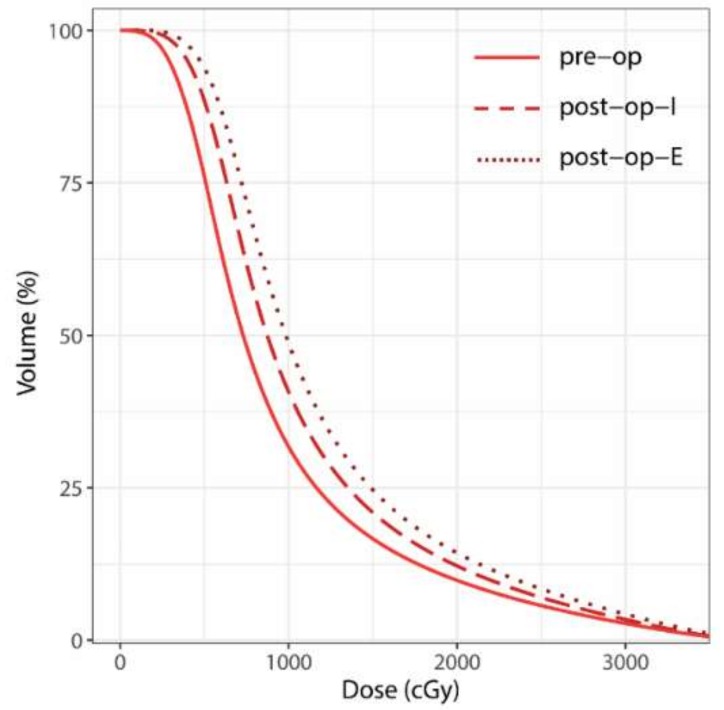
Mean cumulative dose-volume histogram (DVH) curves over all patients for the healthy brain (HB) surrounding the pre-operative and post-operative planning target volumes (PTVs), respectively (= surrounding HB minus respective PTV). Abbreviations: Pre-op: pre-operative; post-op-I: post-operative involved field (cavity + 3 mm); and post-op-E: post-operative extended field (including surgical tract).

No relevant doses were applied to the brain stem, optic system and pituitary gland, consequently no detailed dosimetric analysis was performed for those organs at risk (OAR). In general, dose exposure of the healthy brain (HB) was higher in the post-op settings, compared to the pre-op scenario. Furthermore, dose exposure was generally higher in the post-op-E than in the post-op-I setting, as illustrated in Figure 4.

Regarding median dose/volume value pairs for different dose levels, a trend towards higher dose exposure for post-op-I vs. pre-op setting was observed (*p* ≤ 0.011 for V_1 Gy_ and V_2 Gy_), though in the mid to high-dose range, statistical significance was not reached (*p* ≥ 0.107 for V_5 Gy_ to V_28 Gy_). Dose exposure was significantly higher in the post-op-E setting, compared to the post-op-I setting (*p* ≤ 0.001). This finding suggests a wider spread high and mid-dose distribution when the surgical tract is included in the CTV. Comparing post-op-I and post-op-E, the differences in exposed brain volume were more accentuated in the high-dose range (V_20 Gy_ to V_28 Gy_) than in the mid-dose range (V_5 Gy_ to V_15 Gy_) (Table 4). Interpreted in conjunction with the negligibly small differences regarding conformity and gradient indices (as listed in Table 4), it can be deduced that the differences in dose exposure of the HB were primarily caused by target size and configuration and not by treatment plan quality. Details on dosimetric analysis are displayed in Table 4.

## 3. Discussion

We analyzed locoregional control and survival in 24 patients who received post-operative SRS or HFSRT of the resection cavity at our institution and performed a systematic prospective treatment plan comparison on this cohort. Comparative treatment plans were calculated for a pre-operative and two post-operative scenarios featuring different approaches to margins and contouring. We found the resection cavity to be significantly smaller and different in morphology from the pre-operative lesion. Conversely, including the complete post-operative surgical tract into the CTV only moderately enlarged the resulting target size, as compared to a CTV consisting of only a 3 mm isotropic margin. Dosimetric analysis favored the pre-operative treatment setting since it significantly decreased dose exposure of the healthy brain, as compared to post-operative HFSRT.

### 3.1. Local Control and Patterns of Failure

In our patient collective, distant brain failure was predominant over local failure, and leptomeningeal dissemination was the most common pattern of failure after post-operative SRS/HFSRT of the resection cavity, affecting five out of nine cases (20% overall rate). Three out of five patients developing LMD had breast cancer histology. This finding is in agreement with a growing body of literature that reports the risk of LMD following post-operative SRS to be significantly higher compared to post-operative WBRT (31% vs. 13% at 18 months) or definitive SRS (16.0% vs. 5.2% at 12 months) [12,13,14,15,16,17]. Most of the aforementioned works identified breast cancer histology as a significant risk factor [13,14,15,17]. A presumed pathomechanism for this increased risk of LMD is intra-operative dissemination of tumor cells via the cerebro-spinal fluid, and it is argued that pre-operative SRS could help lower this risk by way of effective sterilization of the resection area [16,18].

Several retrospective series on pre-operative SRS have been recently published, the largest reporting outcome and clinical experience with 117 consecutive patients treated by Prabhu et al. [19,20,21]. They found a cavity local recurrence rate of 25.1% at 2 years, a distant brain failure rate of 60.2% and a comparably low LMD rate of 4.3% after pre-operative SRS at a median dose of 15 Gy [20]. In this latter series, incomplete resection was significantly associated with inferior cavity local control, as well as inferior OS [20].

Several prospective clinical trials are currently ongoing to further examine the effects of treatment sequence, dosage, and fractionation on the oncological outcome: NCT03398694 is a single-arm, phase II trial evaluating pre-operative SRS and surgery for the largest of up to four brain metastases, the rest of which will be treated by SRS only. The authors are additionally planning radiobiological molecular analyses to assess the effect of radiation dosing on the tumor tissue and clinical outcomes [22]. The ESTRON-trial is a randomized phase II trial, evaluating the effect of dose-escalated HFSRT with a total margin width of 4 mm in a post-operative setting, compared to post-operative WBRT [23]. Further trials include NCT03163368 and NCT01252797; both are single-center phase I trials, evaluating maximum tolerated doses for pre-operative SRS.

### 3.2. Volumetric and Dosimetric Comparison

For the pre-operative scenario, we added only a small margin of 1 mm to the macroscopic tumor, as is standard operating procedures at our institution and many other centers similarly [24]. In the post-operative scenarios, the approach to safety margins was based on an expansion of at least 3 mm added to the visible cavity. It can be argued that the margin concepts are fundamentally different between the post-op and pre-op scenarios. In turn, the volumetric differences we found, especially regarding the relative volume increase from GTV to the post-operative PTV, are to a certain extent to be expected. It was our aim in the current analysis to derive the approach to target contouring and margins from the clinical necessity that is associated with the respective scenario. The use of a minimum (mostly 1 mm) or no margin for the SRS of unresected BM is backed by robust clinical experience, showing excellent local control of 80–90% at 12 months with low toxicity [11,25]. In a post-operative scenario however, evidence exists to indicate substantial uncertainties regarding target volume delineation. A systematic prospective evaluation, among other analyses, showed the addition of a 2 mm CTV margin to the visible cavity to significantly improve tumor control [26,27]. However, the results of the recent phase 3 trial by Brown et al. showed significantly inferior local control following cavity SRS compared to WBRT (81% vs. 61% after 12 months, *p* = 0.0007) [7]. These results suggest that a margin of 2 mm, as employed in this trial, might still be insufficient to provide adequate local control. Uncertainties regarding post-operative target delineation are further aggravated by the morphological changes that affect the cavity within the first four to five post-operative weeks, that typically overlap with the period of SRS treatment planning and pre-treatment imaging [26,28]. Several groups have published partly contradicting findings on the topic of post-operative cavity volume changes. Whereas one group found no significant changes in cavity volume between immediate post-operative and delayed treatment-planning imaging [14], others found those changes to be upwards of 2 ccm size reduction or expansion for up to a third of the patients [9]. Those observations were confirmed by a German group, recently analyzing post-operative cavity changes in 57 patients and concluding significant dynamics with cavity shrinkage predominant in 79.1% of the cases [29]. Yet another group found the amount of pre-operative T2 edema to be a significant prognosticator for the probability of post-operative cavity size reduction [30]. Our findings are in agreement with those works suggesting significant changes in cavity volume, since we observed median cavity size to be significantly smaller than the unresected lesion (6.96 ccm vs. 8.71 ccm, *p* = 0.019). In addition, we found the anatomical overlap of the two volumes to be small with a similarity coefficient of only 53%, indicating not only volumetric changes, but also changes in configuration and potential location shifts of the cavity. It is crucial to bear especially the latter in mind when estimating post-operative delineation uncertainties and safety margins. From the discussed data regarding volumetric changes of the cavity we deducted that if a dosimetrical comparison of realistic clinical scenarios is sought, it is warranted to link each scenario with its respective necessity for margin expansion and compare the resulting volumes and their impact on dosimetry. It is this rationale that made us base the pre-operative margin on a 1 mm expansion, whereas the post-operative margin was based on a 3 mm expansion.

Consensus contouring guidelines for postoperative SRS of the resection cavity have recently been compiled [24]. A high level of overall agreement was found for the delineation of the cavity CTV. However, areas of relevant disagreement included the extent to which the surgical tract should be included [24]. By comparing the post-op-I (without surgical tract) and post-op-E (including surgical tract) scenarios, we aimed to evaluate the volumetric and dosimetric impact of including the complete tract, especially with regard to the dose distribution affecting the surrounding healthy brain. The volumetric difference was present, though small, as indicated by the median difference of 2.92 ccm (12.83%) between post-op-I and post-op-E PTV and the high Dice Similarity Coefficient of 93%. We hypothesized, that apart from the potential impact of a larger target volume on surrounding mid and low-dose distribution, the inclusion of the surgical tract could lead to a more complex PTV and negatively impact conformity and gradient indices. Our results showed the effect regarding conformity and gradient indices to be clinically negligible, yielding a median nCI of 1.13 for post-op-I PTV vs. 1.14 for post-op-E PTV. This is possibly due the use of an advanced intensity-modulated irradiation (IMRT) technique that can achieve high conformity for even complex target volumes [31]. However, with IMRT, comparable conformity for increasingly large and complex targets can typically come at the price of a less favorable mid and low-dose distribution [6,32]. This showed in the generally higher dose to the healthy brain in the post-op-E scenario (median V_28 Gy_ (equivalent to V_12 Gy_ in single-session SRS) = 10.79 vs. 8.91 ccm; *p* < 0.001), whereas differences between pre-op and post-op-I were less accentuated.

Several works have associated increasing volumes of healthy brain tissue receiving mid-range doses (e.g., 10–12 Gy) during single-session SRS with an elevated risk of developing radionecrosis [10,33,34]. Assuming an α/β of 2 for neuronal tissue, in a hypofractionated regimen of seven fractions, this would translate into cumulative doses of 23–28 Gy. The healthy brain volumes (total brain minus PTV) receiving doses in this range were the largest in the post-op-E scenario and lowest in the pre-op scenario. Considering that the margin required for pre-operative RT is substantially smaller than in the post-op settings, aforementioned differences in the dose exposure of healthy brain tissue are even more pronounced. From a dosimetrical point of view, those findings decisively favor the pre-operative setting, and they suggest that pre-operative radiotherapy may help lower the risk of radiation necrosis. This is especially true when the complete surgical tract is included in the CTV, as the consensus contouring guidelines recommend [24]. Our findings in this regard are backed by the results of the single available clinical study comparing between pre-op and post-op SRS [8]. Here, the authors found cumulative incidences for symptomatic radiation necrosis of 14.6% (post-op) vs. 1.5% (pre-op) after 1 year and 16.4% vs. 4.9% after 2 years, respectively [8]. In another large retrospective series of 117 patients, a rate of 4.8% after 2 years was found for symptomatic radionecrosis after pre-operative SRS at a median does of 15 Gy [20].

Bearing in mind the issue of radionecrosis, we chose a hypofractionated dose concept in our current analysis. In contrast to single-session SRS, hypofractionation generally allows for the treatment of larger target volumes with a higher dose, as may be crucial in the treatment of a resection cavity [35,36,37]. Assuming an α/β of 10 for malignant cells, the prescribed dose of 7 × 5 Gy translates into a biologically effective dose (BED) of 52.5 Gy. In contrast, the single session doses of 12 to 16 Gy applied to larger cavities in the recent phase 3 trials by Brown et al. and Mahajan et al. translate into a BED of 26 to 41 Gy by the same model, while WBRT at 10 × 3 Gy yields a BED of 39 Gy [7,38]. While the validity of the linear-quadratic model for radiosurgical doses has been challenged, underdosage of SRS probably still played a role in the superiority of WBRT in the phase 3 trials with regard to local control [7,38,39,40,41]. This hypothesis is supported by recently published works reporting more favorable local control rates of up to 88% after 12 months for HFSRT and generally favoring a higher BED [37,42,43]. With fractionated regimens, the risk of radionecrosis can be kept comparably low, reported at 0–4%, while the BED can be escalated [42,44,45]. On the other hand, fractionation prolongs overall treatment time. In consequence, the general feasibility of this approach is disputable with regard to the pre-operative setting when the patient is symptomatic and timely resection indicated.

Another potential limitation of pre-operative radiotherapy is the lack of histologic confirmation. While the risk of falsely treating non-metastatic central nervous system disease has been reported to be extremely low, this aspect renders the pre-operative approach useless for cases where the primary objective of surgery is the procurement of a pathologic sample [3,46,47]. The possibility of peri-operative complications or prolonged wound healing are further potential disadvantages discussed in the context of pre-operative radiosurgery. However, several recent series have not confirmed those reservations, reporting no elevated peri or post-operative complication rate [19,20,21]. Furthermore, in those recently reported series, surgery was generally carried out within 48 hours of SRS completion, choosing an early enough timepoint to avoid radiogenic tissue reactions such as fibrosis or inflammation [20].

Clinical experience shows, that establishing a reliable workflow that allows for pre-operative radiotherapy of brain metastases may require close interdisciplinary communication to overcome organizational obstacles. On the other hand, pre-operative SRS may prove advantageous in a logistical sense. A recent review by Routman et al. named several organizational arguments in favor of the pre-op setting, central among which are a potentially timelier discharge of the patient to rehabilitation and an earlier administration of systemic therapy after resection [48,49]. 

Limitations of our study include its relatively small cohort size. For the clinical endpoints of locoregional control and survival, cohort size restricted the possibility of sound statistical analysis. Sample size estimation, however, was primarily done with respect to the dosimetric and volumetric aspects that were the main objectives of this analysis and for which it was adequate. While the character of our systematic plan comparison is intended to be exploratory, the results lack confirmation in the form of clinical outcome data, and a clinical trial comparing pre vs. post-operative radiotherapy for brain metastases seems warranted. Additionally, potential strengths and limitations regarding our design of the evaluated treatment scenarios with their different approaches to margins and delineation have been discussed above in greater detail.

## 4. Materials and Methods

All analyses were performed following institutional guidelines and the Declaration of Helsinki of 1975 in its most recent version. Ethics approval for the study and a waiver of written informed consent was granted by the Heidelberg University ethics committee on 12 April 2018 (#S-172/2018). Patient confidentiality was maintained by anonymizing patient data to remove any identifying information.

### 4.1. Treatment Planning and Delivery for Performed SRS/FSRT

Patient fixation was accomplished by means of an individually fitted thermoplastic mask. Target delineation and treatment planning was based on a computed tomography (CT) with 1 mm slice thickness and complementary magnetic resonance imaging (MRI). MRI contained a contrast-enhanced, T1-weighted, three-dimensional sequence with multiplanar reconstruction, and a slice thickness of ≤1 mm, which was thoroughly co-registered and served as a basis for target and organs at risk (OAR) delineation. Cavity and residual tumor—if applicable—were contoured as gross target volume (GTV). A total safety margin of 1–3 mm was added by isotropic expansion and slightly adapted by an experienced physician when necessary. Actual treatment planning was done in Accuray’s Multiplan v5.3 (Accuray Inc., Sunnyvale, CA, USA) and subsequent versions, while treatment was delivered using CyberKnife M6 (Accuray Inc., Sunnyvale, CA, USA).

### 4.2. Treatment Planning for Comparative Analysis

Comparative treatment planning, volumetric and dosimetric analysis were performed on RaySearch’s RayStation v6.1.1.2 (RaySearch Laboratories, Stockholm, Sweden), utilizing the comprehensive scripting capabilities for semi-automated plan generation and analysis. Treatment plans were created for HFSRT using volumetric modulated arc therapy (VMAT) with four non-coplanar arcs at a VersaHD™ linear accelerator (Elekta, Stockholm, Sweden) with an Agility™ (Elekta Stockholm, Sweden) multi-leaf collimator (MLC) featuring 5 mm leafs at the isocenter. Prescribed dose for all comparative plans was 35 Gy in 7 fractions to the PTV, aiming for a coverage of at least 98%, The prescribed dose was scaled to the conformally surrounding 70% isodose (maximum = 100%).

To evaluate the volumetric and dosimetric impact of different treatment settings (pre vs. post-operative) and margin concepts, three hypothetical clinical scenarios were constructed and compared for each patient. Target volume definition and treatment planning for those hypothetical scenarios were done independently from the de facto performed treatment in order to achieve a homogeneous sample with regard to delineation and treatment planning strategy. Comparative plans were not actually used for treatment. Target delineation with strict adherence to the specifications outlined below was performed by a radiation oncologist with extensive experience in intracranial radiosurgery. All target volumes were then reviewed and approved by a second experienced radiation oncologist and differences were resolved by individual discussion. The evaluated treatment scenarios are illustrated in Figure 1 and were defined as follows:

#### 4.2.1. Pre-Operative (pre-op)

The pre-operative MRI was registered with the post-operative treatment-planning CT to delineate the location and extent of the resected lesion. For a hypothetical pre-operative HFSRT, a planning target volume (PTV) margin of 1 mm by isotropic expansion was added to the macroscopic tumor, as is the institutional standard for the SRS/HFSRT of unresected brain metastases.

#### 4.2.2. Post-Operative Involved Field (post-op-I)

On the post-operative MRI, the resection cavity and—if applicable—residual tumor without an additional margin and without the surgical tract were defined as GTV. The clinical target volume (CTV) was created by adding an isotropic margin of 3 mm to the GTV. As above, for PTV creation, an additional margin of 1 mm was added by isotropic expansion.

#### 4.2.3. Post-Operative Extended Field (post-op-E)

GTV delineation was performed as in post-op-I. The CTV was created by adding an isotropic margin of 3 mm to the GTV and—if necessary—enlarging the resulting structure to encompass the complete surgical tract and all contrast-enhancing parenchymal and meningeal tissue associated with it. As above, for PTV creation, an additional margin of 1 mm was added to the CTV.

### 4.3. Volumetric and Dosimetric Analysis

GTV volumes were compared between the pre-operative and post-operative setting and overlap, as well as difference between the post-op and pre-op GTV were calculated using RayStation’s region of interest (ROI) algebra functionality to quantify the post-operative morphological change of the cavity. Furthermore, the absolute and relative increase in volume from GTV to PTV, due to the application of the aforementioned differing margin concepts, was calculated and compared between all three clinical scenarios. The Dice Similarity Coefficient (DSC) was calculated using RayStation’s geometry comparison functionality between 1) pre-op GTV and post-op GTV and 2) post-op-I PTV and post-op-E PTV, respectively. The DSC, as described by Sørensen and Dice et al., can be utilized to quantify morphological similarity between two contours, or in more general terms, two sets of data, and is defined as the quotient of the intersection and sum of both data sets, as follows [42,43]:

(1) $$DSC=\frac{2\times \left|X\cap Y\right|}{\left|X\right|+\left|Y\right|}$$

Herein |X| and |Y| represent the number of voxels in each set/contour. A higher DSC (given in %) describes high similarity/overlap between two samples, while a lower value describes outweighing differences [42,43].

For each of the abovementioned three scenarios, the healthy brain (HB) contour was created by subtracting the respective PTV from the whole brain contour. The brain volume receiving 28, 25, 20, 15, 10, 5, 2, 1 (V_28 Gy_, V_25 Gy_, V_20 Gy_, V_15 Gy_, V_10 Gy_, V_5 Gy_, V_2 Gy_, V_1 Gy_) were determined and compared between plans. Cumulative dose-volume histogram (DVH) curves were calculated for the HB. Dose conformity to the PTV was assessed using the new conformity index (nCI), defined as the reciprocal of the modified Paddick Conformity Index as follows [44,45]:

(2) $$nCI=\frac{V_{PTV}\times V_{pi}}{{\left(V_{PTV,pi}\right)}^{2}}$$

The nCI is a commonly employed quality parameter in radiosurgery; it assesses the quality of target coverage, as well as the dose to surrounding healthy tissue, where V_PTV_ is the planning target volume, V_pi_ is the body volume of the patient covered by the prescribed dose and V_PTV, pi_ is the partial volume of the PTV covered by the prescribed dose [44,45].

To assess dose falloff outside the target volume, two gradient indices, as described by Paddick et al., and modified by Stieler et al. were calculated:

GI_high_ = V_50%Presc.Dose_/V_90%Prescr.Dose_ (3)

and [46,47]

GI_low_ =V_25%Presc.Dose_/V_50%Prescr.Dose_ (4)

### 4.4. Statistical Analysis

Descriptive statistics are used for baseline analyses; continuous variables are given as median (IQR and range, mean and standard deviation, as appropriate) and categorical variables as absolute and relative frequencies. The Kaplan-Meier method was used to investigate locoregional control after performed treatment, more specifically progression-free survival at the cavity (cPFS) and in the distant brain (dPFS), and overall survival (OS). Locoregional control was calculated from the time of treatment to the last available imaging follow-up or progression. Overall survival was calculated from the time of treatment to the last available clinical follow-up or death. The median follow-up time was calculated using the reverse Kaplan-Meier method [48]. Regarding the volumetric and dosimetric analyses, normality assumption may be violated due to the small sample size, and therefore non-parametric statistical methods were used. All method comparisons were done by the Wilcoxon signed rank test for paired data. DVH curves were generated with the DVHmetrics package for R [49]. Since this was primarily an experimental analysis, *p*-values are attributed no confirmatory character. A descriptive *p*-value of ≤0.05 was considered statistically significant. Statistical analyses were performed with the software R version 3.5.1. (www.r-project.org).

## 5. Conclusions

To the best of our knowledge, this analysis is the first to assess the volumetric and dosimetric impact of pre vs. post-operative treatment setting and margin size for the radiosurgery of brain metastases by means of a systematic treatment plan comparison. It supports the hypothesis that including the extent of safety margin that seems necessary to achieve adequate local control, significantly enlarges the resulting post-operative target volume. We furthermore detected substantial post-operative morphological changes regarding cavity size and configuration, heightening delineation uncertainty, and further justifying an increase in safety margin size. The hence resulting increase in dose exposure of the healthy brain and the implications for the risk of radiation toxicity favor the pre-operative treatment setting. However, confirmatory clinical trials are required to further validate this line of reasoning.

## Figures and Tables

**Table 1:** Patient characteristics (*n* = 24).

Characteristics	*n*	%
**Age at Radiotherapy (years)**	
Median	60	
Q1-Q3	52–64	
**Gender**	
female	16	66.67%
male	8	33.33%
**Additional Unresected Brain Metastases**	
0	14	58.33%
1	5	20.83%
2	4	16.67%
4	1	4.17%
**Primary Histology**	
NSCLC	9	37.5%
breast cancer	7	29.17%
renal cell carcinoma	3	12.5%
upper gastrointestinal tract	2	8.33%
other	3	12.5%
**Location of Resected Brain Metastasis**	
supratentorial	16	66.67%
infratentorial	8	33.33%

NSCLC: non-small cell lung cancer.

**Table 2:** Detailed treatment parameters for performed treatment.

Detailed Treatment	*n*	%
**Interval Between Surgery and Radiotherapy (days)**	
Mean	60	
Median	40	
SD	72	
Q1–Q3	35–48	
Min.–Max.	23–377	
**treatment modality**	
HFSRT	16	66.67%
SRS	8	33.33%
**width of safety margin (mm)**	
1	6	25%
2	3	12.5%
3	15	62.5%
**complete resection canal included in GTV**	
no	13	54.17%
yes	11	45.83%
**physical dose for SRS (*n* = 8)**	
18	4	50%
20	2	25%
12	1	12.5%
17	1	12.5%
**cumulative physical dose for HFSRT (*n* = 16)**	
Mean	31	
Median	30	
SD	NA	
Q1–Q3	30–31	
Min.–Max.	30–35	
**number of fractions for HFSRT (*n* = 16)**	
Mean	6	
Median	6	
SD	NA	
Q1–Q3	6–6	
Min.–Max.	6–7	
**biologically equivalent cumulative dose for SRS/HFSRT (α/β = 10)**	
Mean	48	
Median	45	
SD	6	
Q1–Q3	45–51	
Min.–Max.	26–60	
**prescription isodose for SRS/HFSRT (%)**	
Mean	77	
Median	70	
SD	8	
Q1–Q3	70–86	
Min.–Max.	65–88	
**dose exposure of the healthy brain for SRS (*n* = 16)**		**median (Q1–Q3) ccm**
V_8 Gy_		23 (21–38)
V_10 Gy_		15 (14–23)
V_12 Gy_		10 (8–12)
V_14 Gy_		7 (5–8)
V_16 Gy_		4 (2–5)
**dose exposure of the healthy brain for HFSRT (*n* = 8)**		**median (Q1–Q3) ccm**
V_15 Gy_		48 (35–56)
V_18 Gy_		33 (24–39)
V_21 Gy_		24 (17–27)
V_24 Gy_		16 (10–19)

GTV: gross target volume; SRS: stereotactic radiosurgery; HFSRT: hypofractionated stereotactic radiotherapy; V_X Gy_: volume receiving X Gy; SD: standard deviation.

**Table 3:** Morphological comparison of target volumes and margins, including similarity, areas of overlap and difference. Absolute volumes are listed to the left; to the right are listed volumes relative to the combined sum of the two respective targets.

Morphological Comparison	ccm		% (Relative to Sum)	
	median	Q1–Q3	median	Q1–Q3
**cavity morphology (pre-op vs. post-op)**				
overlap GTV pre-op and cavity post-op	3.98	2.31–13.69	36.05%	29.46–46.26%
DSC GTV pre-op and cavity post-op	-	-	53%	46–63%
post-op not in pre-op	1.49	0.84–3.28	14.14%	5.85–27.89%
pre-op not in post-op	4.34	2.77–11.52	43.50%	24.14–51.35%
**effect of added margins: PTV (post-op-I vs. post-op-E)**				
overlap PTV post-op-I and post-op-E	18.53	13.88–31.12	86.31%	77.54–89.27%
DSC PTV post-op-I and post-op-E	-	-	93%	87–94%
post-op-I not in post-op-E	0.01	0.00–0.07	0.05%	0.00–0.27%
post-op-E not in post-op-I	2.92	2.14–4.84	12.83%	10.34–21.68%

Abbreviations: GTV: gross target volume; PTV: planning target volume; pre-op: pre-operative setting; post-op-I: post-operative involved field (cavity + 3 mm); post-op-E: post-operative extended field (including surgical tract); and DSC: Dice Similarity Coefficient.

**Table 4:** Detailed quantitative results of volumetric and dosimetric comparison between the pre-operative (pre-op) and two post-operative settings, targeting the involved field (cavity + 3mm, post-op-I) and extended field including the complete surgical tract (post-op-E). To the right, p-values for pair-wise comparison (Wilcoxon signed rank test for paired data) are displayed. Significant p-values are indicated in bold type.

Detailed Results	pre-op	post-op-I	post-op-E	post-op-I vs. pre-op	post-op-E vs. pre-op	post-op-I vs. post-op-E
	median (Q1–Q3)	median (Q1–Q3)	median (Q1–Q3)	*p*	*p*	*p*
**volume (ccm)**						
GTV	8.71 (5.47–26.08)	6.96 (4.16–15.50)	6.96 (4.16–15.50)	**0.019**	-	-
CTV	-	15.29 (11.32–27.69)	18.96 (12.86–31.75)	-	-	**<0.001**
PTV	12.29 (8.13–30.20)	18.58 (13.88–31.30)	22.89 (16.37–37.21)	**0.016**	**0.002**	**<0.001**
**volume increase**						
GTV to PTV (absolute (ccm))	2.47 (1.70–4.35)	12.14 (9.31–16.57)	15.9 (11.52–22.87)	**<0.001**	**<0.001**	**<0.001**
GTV to PTV (relative to GTV (%))	23.75% (15.87–30.36%)	175.91% (107.25–221.50%)	224.41% (139.49–290.27%)	**<0.001**	**<0.001**	**<0.001**
**dose to the healthy brain (median volume in ccm)**						
V_28 Gy_	6.79 (4.28–12.79)	8.91 (6.01–11.08)	10.79 (7.01–13.54)	0.345	**0.005**	**<0.001**
V_25 Gy_	10.14 (6.25–18.73)	13.39 (8.68–18.96)	15.38 (9.94–19.93)	0.229	**0.004**	**<0.001**
V_20 Gy_	18.07 (10.76–31.79)	23.21 (14.71–32.86)	26.69 (16.43–34.08)	0.188	**0.006**	**<0.001**
V_15 Gy_	31.24 (18.09–53.53)	39.25 (25.61–56.81)	46.07 (27.61–58.68)	0.178	**0.005**	**0.001**
V_10 Gy_	61.42 (33.60–105.51)	77 (51.45–109.33)	88.64 (52.93–118.15)	0.114	**0.003**	**<0.001**
V_5 Gy_	174.35 (91.39–270.16)	203 (142.62–291.11)	227.78 (139.79–313.24)	0.107	**0.003**	**<0.001**
V_2 Gy_	543.38 (370.01–738.26)	637.6 (485.45–778.40)	669.7 (489.51–856.66)	**0.011**	**<0.001**	**<0.001**
V_1 Gy_	858.48 (646.83–927.82)	875.18 (745.86–1007.22)	974.05 (850.44–1071.30)	**0.009**	**<0.001**	**<0.001**
**gradient and conformity indices**						
GI_high_	2.52 (2.22–2.89)	2.38 (2.26–2.58)	2.43 (2.22–2.56)	**0.010**	0.065	0.229
GI_low_	2.6 (2.48–2.71)	2.46 (2.38–2.57)	2.42 (2.35–2.50)	**0.025**	**0.001**	**0.021**
nCI (PTV)	1.12 (1.10–1.19)	1.13 (1.09–1.14)	1.14 (1.13–1.18)	**0.039**	**0.004**	**<0.001**

Abbreviations: GTV: gross target volume; CTV: clinical target volume; PTV: planning target volume; V_X Gy_: volume receiving X Gy; GI_high_: gradient index high; GI_low_: gradient index low; nCI: new conformity index.
